# Incidence of Avascular Necrosis of the Femoral Head Post Hip Reduction Surgery in Children With Cerebral Palsy

**DOI:** 10.7759/cureus.75348

**Published:** 2024-12-08

**Authors:** Edidiong Essiet, Chinedu Egu, Samuel Akintunde, Mohammed Qasim Rauf

**Affiliations:** 1 Trauma and Orthopaedics, Royal National Orthopaedic Hospital, London, GBR; 2 Spinal Surgery, Queens Medical Centre, Nottingham University Hospitals, Nottingham, GBR; 3 Spinal Studies and Surgery, Queens Medical Centre, Nottingham University Hospitals, Nottingham, GBR; 4 Orthopaedic Surgery, Hillingdon Hospital, London, GBR

**Keywords:** avascular necrosis of the femoral head, cerebral palsy, hip reduction, hip reduction surgery, incidence, osteonecrosis of the femoral head

## Abstract

Avascular necrosis (AVN) of the femoral head confers a risk of morbidity amongst cerebral palsy (CP) patients. Meanwhile, the proportion who develop AVN post hip reduction surgery is unclear, and the risk factors are not established. To estimate the incidence and risk factors for AVN post hip reduction surgery in CP patients, we reviewed and analysed literature from Medline, Embase, and Web of Science repositories between January 1, 1990, and August 31, 2024. The included publications were reviewed for the incidence and risk factors in this cohort of patients. Findings were appraised using the methodological index for non-randomised studies, and scores and results were descriptively analysed. Given the marked heterogeneity of retrieved data, results were reported as standardised values, including the mean weighted incidence rate. A total of 1619 hip reduction surgeries were performed on 1045 CP patients across 12 retrospective studies with 424 cases of AVN. The mean age at surgery ranged from 7.3 to 16.2 across studies. The mean incidence was 0.482% ± 0.06945 per person-year. Most patients were in gross motor function classification system V, with a preoperative mean Reimers index of 54.11-80%. The average methodological items for non-randomised studies score was 10.08/16 ± 0.28 for all studies (n=12). AVN has a definite relationship with hip reduction surgery. The low methodological quality of reviewed studies informs the need for well-designed prospective randomised controlled studies to understand the mechanics of this relationship in terms of age of surgery and Reimers hip migration index, guide surgical timing, and patient selection.

## Introduction and background

Cerebral palsy (CP) denotes a group of enduring movement disorders manifesting in early childhood. Hip dislocation is a prominent concern amongst the complications associated with CP, approaching 28%, particularly in gross motor function classification system (GMFCS) IV and V patients [1]. Consequently, hip reduction surgery, including pelvic and femoral osteotomies in conjunction with muscle release, has emerged as a standard of care [2]. Nonetheless, a potential complication arising post-procedure is avascular necrosis (AVN), which presents significant challenges in patient outcomes and management [3]. This review seeks to investigate the incidence of AVN in patients undergoing hip reduction surgery in the subset of patients with CP.

The demise of bone tissue characterises AVN due to a compromise of vascular supply. Within the context of hip reduction surgery in children with CP, the rates of AVN incidence have garnered considerable interest. Various studies have reported divergent rates of AVN following hip reduction surgery, ranging from as low as 10% to as high as 50%. Such variations are contingent upon factors such as patient age, severity of hip displacement, surgical technique, and postoperative care protocols [4].

Numerous risk factors have been identified for the development of AVN following hip reduction surgery in children with CP. These encompass the severity of preoperative hip displacement, the presence of associated co-morbidities such as spasticity and contractures, prolonged intraoperative traction, excessive surgical manipulation, and inadequate postoperative rehabilitation [4-5]. Furthermore, younger age at the time of surgery has been linked to a heightened risk of AVN [6], conceivably owing to the increased susceptibility of the immature femoral head to vascular insult [7].

The clinical presentation of AVN post hip reduction surgery in children with CP can exhibit substantial variability, encompassing asymptomatic radiographic findings to severe pain and functional impairment. Common symptoms are hip pain, restricted range of motion, and gait abnormalities. Diagnosis typically entails a blend of clinical assessment, radiographic imaging such as X-rays and MRI, and evaluation of vascular supply to the affected area utilising techniques like bone scintigraphy or Doppler ultrasound [8].

The management of AVN-ensuing hip reduction surgery in children with CP often presents significant clinical hurdles. Initially, conservative measures such as activity modification, physical therapy, and analgesia may be employed for asymptomatic or early-stage cases. However, advanced instances may necessitate surgical intervention, encompassing core decompression, bone grafting, or total hip arthroplasty, contingent upon the extent of bone involvement and patient age [9-10]. Despite advancements in surgical techniques and rehabilitation protocols, the prognosis for AVN remains variable, influenced by factors such as the timing of diagnosis, the extent of bone involvement, and the effectiveness of treatment interventions [11].

AVN represents a significant complication following hip reduction surgery in children with CP, with reported incidence rates exhibiting wide variance across studies. For example, an incidence of 68.7% was identified by Koch et al. with significant links between necrosis and multiple risk factors like the shape of the femoral head, surgery conducted after age 8, and a neck-shaft angle below 140 [12]. Understanding risk factors, clinical presentation, diagnosis, and management strategies for AVN is imperative for optimising patient outcomes and guiding clinical decision-making in this demographic. Thus, we systematically reviewed the literature from 1990 to date to determine the incidence and prevalence of AVN in CP patients.

## Review

Materials and methods

Preparatory to conducting this study, a detailed systematic review protocol was registered on the International Prospective Register of Systematic Reviews (CRD42023412616): https://www.crd.york.ac.uk/prospero/display_record.php. RecordID=412616 and published. Thus, only a summary of the methods related to the review of epidemiology is provided here. The review method was formulated with adherence to the Preferred Reporting Items for Systematic Reviews and Meta-analyses (PRISMA) [13].

Search Strategy

An extensive electronic literature search was conducted to identify English language publications on the subject between January 1, 2000, and August 31, 2024, as indexed in Embase, Medline (PubMed), and Web of Science. The strategy was to search, by use of the Boolean principle, for the presence or absence of synonyms of “avascular necrosis of the femoral head”, “hip reduction”, and “cerebral palsy”.

(avascular necrosis OR osteonecrosis OR aseptic necrosis OR ischaemic bone necrosis) AND (hip reduction OR hip surgery OR open reduction of the hip OR closed reduction of the hip) AND (femoral head OR head of the femur) AND (cerebral palsy).

No terms for incidence were included in the search to ensure that all studies on AVN of the femoral head were captured. Furthermore, two independent reviewers hand-searched bibliographies of selected articles and reviews relevant to the question.

The search was limited to articles written in English, as including other languages would not significantly affect the outcome. Also, letters, commentaries, editorials, opinion papers, education papers, case reports, and book chapters were excluded, as were further studies on AVN of the femoral head whose population does not include patients with CP or does not report outcomes of interest. No restrictions were applied on the number of patients included in the study or the type of study. Monthly email alerts were set up for each database to ensure that content accurately reflects research reported within the review's proposed time frame. A forward citation search using Google Scholar was performed to capture early online articles published since January 1st, 2024. Obtained, eligible results were planned for inclusion through the data extraction phase.

Data Management

Search results from the three databases were exported into the Rayyan systematic review and meta-analysis software (Qatar Computing Research Institute, Doha, Qatar), and duplicates were removed. The full text of the remaining articles was retrieved and linked to the corresponding record on Rayyan software. Studies were selected on the Rayyan software and information exported to Excel 2020 (Microsoft Corporation, Redmond, WA, USA). The spreadsheet was expanded and applied for data extraction and critical analysis.

Inclusion Criteria

Children under 18 years of age with CP who underwent hip reduction were included. Inclusion criteria were independent of gender, study location, ethnicity, symptomatology, outcomes, or type of CP. Studies were selected if they were primary research on AVN of the femoral head following hip reduction in children with CP. The studies included could be observational, interventional, or peer-reviewed. Studies that reported the incidence of AVN of the femoral head categorised patients with CP and stratified them based on age were included.

Exclusion Criteria

Studies not published in English, as well as editorials, commentaries, opinion papers, letters, educational papers, conference abstracts, protocols, reports, book chapters, or laboratory-based studies, will not be included. Additionally, studies on AVN that do not include patients with CP or respondents over 18 years of age will be excluded.

Appraisal of Quality

Most retrieved studies were mainly observational or non-randomised, so the methodological index for non-randomised studies (MINORS) [14] was used to appraise the methodological quality and categorise studies based on their strengths. The risk of bias in the included studies was assessed independently by two reviewers using the MINORS tool for randomised studies. The criteria were "0" if not reported, "1" when reported but inadequate, and "2" when reported and adequate. The general ideal score was 16 for non-comparative studies and 24 for comparative studies. The MINORS score was provided for each study to enable readers to access documented evidence as indisputable, unsupported, or credible.

Data Extraction

A proforma will be designed to collect systematic data extraction with information on (a) author, (b) type of study, (c) follow-up period (years), (d) age, (e) gender, and (f) type of CP.

Reviewers extracted data from all selected literature to ensure the reliability of the collection. Discrepancies were discussed, and an independent collaborator to facilitate decision-making was required. Extracted data was recorded using Rayyan systematic review software. Each article was independently appraised, and efforts were made to contact the authors of incomplete articles, and reminders were sent when responses were not obtained. For articles where data for the same participants were reported, subject numbers, demographics, and type of surgery were compared across studies for discrepancies. Any uncertainty about the similarity in study participants and results was clarified by efforts at contacting the authors of the original research. Where the same subjects were included in multiple studies, data were treated as a single source, but all studies were cited.

Data Reporting

Extracted data was examined to identify variations in how the incidence rate was reported, and, where possible, efforts were made to reduce apparent variations. We reported the mean incidence of studies and expressed this in person years as a percentage to reduce variance and facilitate synthesis. As part of the narrative review, internal and external validity issues that most influenced the incidence rates or prevalence were discussed with a specific focus on limitations that lead to imprecision and heterogeneity [15].

Where possible, descriptive statistics were used to summarise data. A mean annual incidence rate was calculated using the crude incidence rates for each included study. Further, considering that the same individual could have bilateral AVN resulting from two surgical procedures occurring at different times, the rates in each study were weighted by the mean number of reported diagnoses per annum to further reduce variance and improve accuracy.

Data Analysis

Data analysis of findings was mainly predominantly descriptive, as the numerical data obtained were neither uniform nor complete. A number of articles were deficient in metrics relevant to the aims of this study. The number of included studies differed across metrics. To calculate the incidence rate, the number of patients was multiplied by the total follow-up period to obtain the total number of patient-years. The number of patients with AVN was then divided by the number of patient years to obtain the incidence rate. Due to the wide variability of sample sizes across studies, a random effect model was used to estimate the cumulative incidence risk, weighted by the average number of reported cases of AVN per year, to generate a point estimate and 95% confidence interval using Stata version 16.0 (StataCorp LLC, College Station, TX, USA). Subgroup analysis based on age and gender was impossible due to the unavailability of raw data from the included studies.

Results

**Figure 1:**
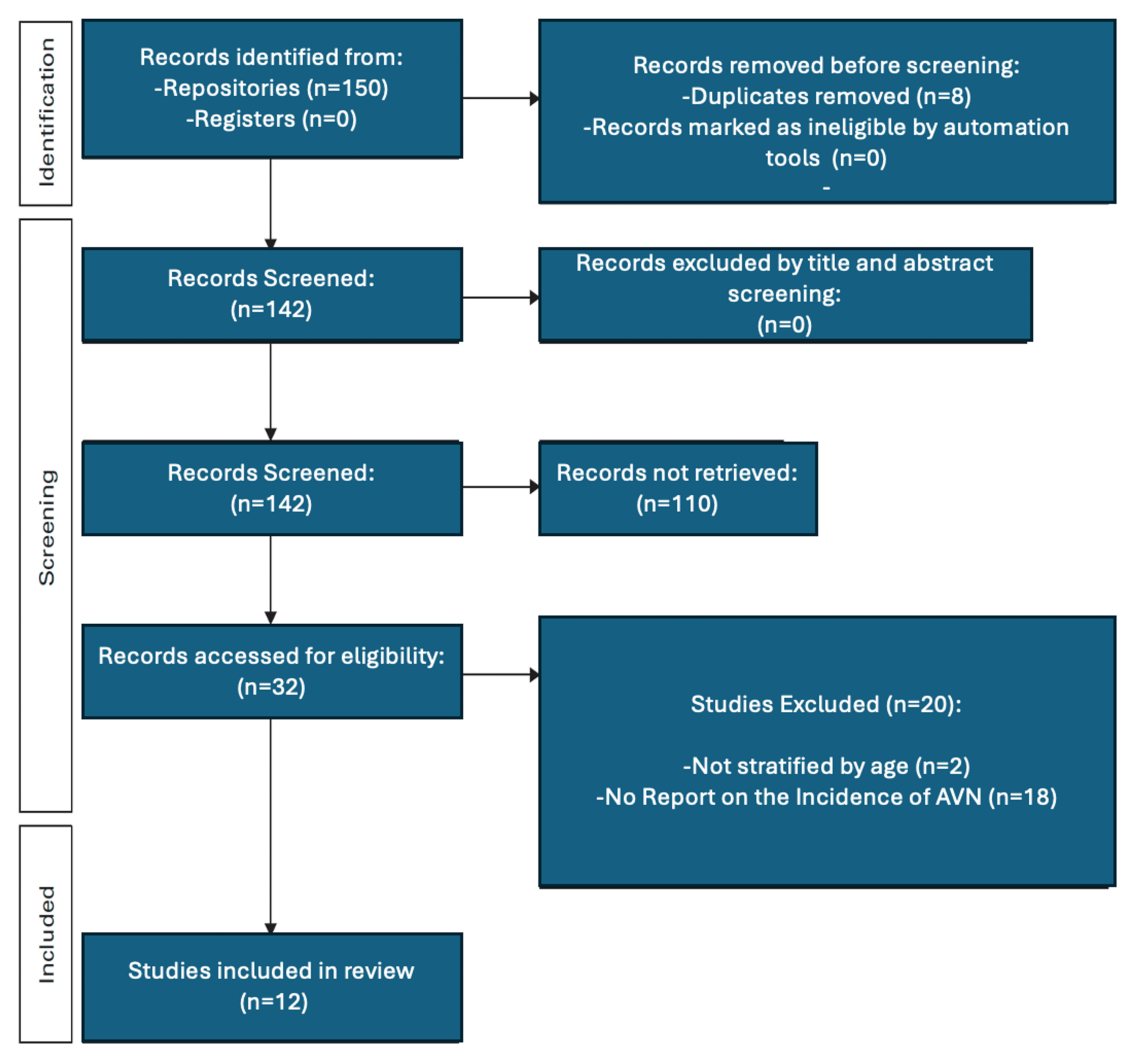
PRISMA flowchart showing steps in the research process PRISMA: Preferred Reporting Items for Systematic Reviews and Meta-analyses, AVN: avascular necrosis

The initial search produced 150 results. Upon removal of duplicates, the figure dropped to 142 articles, which were further matched against the inclusion criteria based on their titles and abstracts. Thirty-two articles were available for full-text review, 12 of which fulfilled the inclusion criteria (Figure 1). A hand search of the reference list of these articles did not produce any additional results. Forward citation searching revealed no additional articles.

Characteristics of Included Studies

Studies included in the review were spread across 10 countries: the USA and Korea accounted for five (45%) of the included studies (Table 1). All included studies were cohort designs and employed retrospective data from paediatric orthopaedic clinics or hospital databases. Classifying hospitals based on care levels was difficult because terminology differences necessitated intimate knowledge of the settings for accurate categorisation. None of the studies collected prospective data.

**Table 1:** Characteristics and demographics of included studies

Author	Country	Year	Patients (n)	Sex		Hips (n)	Hips with AVN (n)	Mean follow-up (years)
				M	F			
McNerney et al. 2000 [19]	USA	2000	75	44	31	104	8	6.9 years
Khalife et al. 2010 [16]	Lebanon	2010	50	17	33	89	33	5 years
Kim et al. 2012 [21]	Korea	2012	23	13	10	32	2	2.3 years
Canavese et al. 2013 [22]	Argentina	2013	24	15	9	30	3	3 years
Koch et al. 2015 [12]	Poland	2015	81	40	41	115	79	5.5 years
Canavese et al. 2017 [24]	France, Switzerland	2017	54	34	20	64	0	3.6 years
Phillips et al. 2017 [20]	Canada	2017	47	31	16	70	19	4 years
da Silva Gomes et al. 2022 [18]	Brazil	2022	104	69	35	128	62	5 years
Park et al. 2022 [6]	Korea	2022	205	120	85	394	169	5.6 years
Bauer et al. 2022 [4]	USA	2022	154	93	61	223	38	6.5 years
Minaie et al. 2022 [17]	USA	2022	209	116	93	349	11	3.5 years
Chen et al. 2022 [23]	China	2022	19	11	8	21	0	3.4 years

The proportion of children with AVN post hip reduction surgery in the included studies varied significantly, possibly because of the wide variation in the time frame (median duration of 4.79 years; range 2.3-6.9 years) and the degree of specialisation of the hospitals. The total cohort size of all included studies was 1619 hip surgeries on 1045 patients (M 603, F 442), with 424 hips developing radiologic signs of AVN (Table 1). The mean age at surgery was 9.2 ± 2.2 years. Amongst studies that reported on the GMFCS classification, most patients were GMFCS V (n=381) and GMFCS IV (n=212). Two papers [6,16] did not classify patients based on their GMFCS class. Most studies captured a cohort size larger than 100 hip surgeries in CP patients and demonstrated an average of >30 cases of AVN per study [4,6,12,16-20]. In contrast, two studies demonstrated a population of approximately 30 hip surgeries with three and two cases of AVN, respectively [21,22]. Two studies reported no cases of AVN amongst 21 [23] and 64 hips [24].

Concerning the selection criteria, all studies reported on a population of CP paediatric patients who underwent hip reduction surgery and developed AVN. Data on the procedure performed was excluded due to variability in procedures, surgical approach, and soft tissue release inclusion across the included studies. The mean preoperative Reimers index ranged from 54.11% to 68.70% from 10 included studies (Table 2). Two studies [16,17] did not provide representable recordings of the mean Reimers index of included respondents.

**Table 2:** Mean incidence rate of included studies in patient-years AVN: avascular necrosis, SD: standard deviation

Author	Duration	Sample size (n)	Hips with AVN (n)	Mean age at surgery	Mean incidence/per person-year	
					Mean (%)	SD
McNerney et al. 2000 [19]	6.9 years	104	8	8.1	0.11	0.278
Khalife et al. 2010 [16]	5 years	89	33	7.4	7.42	0.336
Kim et al. 2012 [21]	2.3 years	32	2	8.6	2.72	0.0192
Canavese et al. 2013 [22]	3 years	30	3	9.4	3.33	0.1824
Koch et al. 2015 [12]	5.5 years	115	79	9	12.5	0.331
Canavese et al. 2017 [24]	3.6 years	64	0	9.1	0	0
Phillips et al. 2017 [20]	4 years	70	19	8.8	6.79	0.26
da Silva Gomes et al. 2022 [18]	5 years	128	62	10	9.69	0.0117
Park et al. 2022 [6]	5.6 years	394	169	7.3	7.66	0.0057
Bauer et al. 2022 [4]	6.5 years	223	38	8.5	2.62	0.0239
Minaie et al. 2022 [17]	3.5 years	349	11	8.3	0.90	0.0014
Chen et al. 2022 [23]	3.4 years	21	0	16.2	0	0

Incidence of AVN Post Hip Reduction Surgery in CP Patients

Data on the epidemiology of AVN in our population of interest was reported, as well as the number of cases post-surgery with radiological signs under the surveillance period. The cumulative mean incidence is 0.00482 (0.482%) ± 0.06945 per person-year. Calculating the rate by age and sex was not feasible because data on this categorisation was incomplete across included studies. All studies included in this review used retrospectively collected data. All 12 (100%) of the included studies had clear aims and outcomes. It was difficult to stratify the incidence and risk factors based on the GMFCS classification, Reimers index, and the procedure performed due to the variability of reported variables in included studies.

Quality Assessment

All studies included in this review used retrospectively collected data and were non-comparative studies. All studies had clear aims and outcomes. The MINORS score for each study is shown in Table 3 and ranges from 10 to 11 (of a maximum of 16) for included studies. No study reported on a sample size calculation, and statistics were mainly descriptive, assessing incidence using cumulative and occasionally fixed effect models. The mean MINORS score was 10.08 ± 0.28.

**Table 3:** Appraisal of selected studies using the MINORS score MINORS: methodological index for non-randomised studies

	da Silva Gomes et al. 2022 [18]	Park et al. 2022 [6]	Koch et al. 2015 [12]	Khalife et al. 2010 [16]	Canavese et al. 2017 [24]	McNerney et al. 2000 [19]	Kim et al. 2012 [21]	Bauer et al. 2022 [4]	Canavese et al. 2013 [22]	Minaie et al. 2022 [17]	Phillips et al. 2017 [20]	Chen et al. 2022 [23]
A clearly stated aim	2	2	2	2	2	2	2	2	2	2	2	2
Inclusion of consecutive patients	2	2	2	2	2	2	2	2	2	2	2	2
Prospective collection of data	0	0	0	0	0	0	0	0	0	0	0	0
Endpoints appropriate to the aim of the study	2	2	2	2	2	2	2	2	2	2	2	2
Unbiased assessment of the study endpoint	1	1	1	1	1	1	1	1	1	1	1	1
Follow-up period appropriate to the study aim	2	2	2	2	2	2	2	2	2	2	2	2
Loss to follow up less than 5%	1	1	1	1	1	1	2	1	1	1	1	1
Prospective calculation of the study size	0	0	0	0	0	0	0	0	0	0	0	0
Total	10/16	10/16	10/16	10/16	10/16	10/16	11/16	10/16	10/16	10/16	10/16	10/16

Discussion

The systematic review of AVN following hip reduction surgery in CP patients highlights significant variations, with reported rates ranging from 0% to >50%, a prevalence of 26.2% and a mean incidence of 0.482% per person-year. The variability reflects differences in patient demographics, the severity of preoperative hip displacement, surgical techniques, and follow-up periods. These findings align with existing literature, which similarly documents a wide range of AVN incidence rates post-surgery, influenced by patient factors and surgical procedures.

One key factor contributing to the development of AVN is the age at surgery. Younger patients, particularly those under the age of 8, are at higher risk due to the increased susceptibility of the immature femoral head to ischaemic insult [25]. This review identified a mean age of 9.2 years across studies, suggesting a patient population at considerable risk. The findings support the recommendation for prudent patient selection and consideration of age when planning surgical interventions, as highlighted by Koch et al., who reported an increased incidence of AVN in patients undergoing surgery after age 8.

Preoperative measures, such as the Reimers hip migration index, were frequently reported in the included studies, with indices ranging from 54.11% to 80%. A higher Reimers index indicates greater hip displacement, which is associated with increased surgical complexity and a heightened risk of AVN. The included studies predominantly featured patients classified as GMFCS IV and V, correlating with severe motor impairment and increased hip instability. This aligns with previous findings that GMFCS classification significantly predicts hip displacement and subsequent AVN risk [26]. The review underscores the challenges in standardising surgical techniques across studies, as variability in procedures (e.g., inclusion of soft tissue release) affects the outcomes and complicates comparisons. The lack of prospective studies in the current review further highlights this gap, as all included studies relied on retrospective data.

Management of AVN post-surgery remains complex, often requiring a combination of conservative and surgical interventions. Early-stage AVN may be managed with rehabilitation and analgesia, while advanced cases may necessitate procedures like core decompression or total hip arthroplasty [27]. Despite advancements in these treatments, the prognosis for AVN remains guarded, with outcomes highly dependent on the timing of diagnosis and the extent of femoral head involvement. The methodological quality of the included studies, as assessed by the MINORS score, was relatively low, with a mean score of 10.1 out of 16. This reflects the predominance of observational, non-randomised studies, which confers a risk of bias. The review emphasises the need for well-designed prospective randomised controlled trials to provide robust evidence on AVN risk factors and incidence that could guide patient selection and approaches to management.

## Conclusions

AVN represents a significant complication following hip reduction surgery in children with CP, with incidence rates influenced by patient age, preoperative hip displacement, and surgical technique. The current evidence underscores the importance of careful patient selection, standardised surgical approaches, and early diagnosis to mitigate the risk of AVN. Future research should focus on high-quality prospective studies to elucidate the mechanisms underlying AVN development and inform best practices for surgical timing and technique.
